# Draft Genome Sequence of *Clostridium perfringens* Strain JJC, a Highly Efficient Hydrogen Producer Isolated from Landfill Leachate Sludge

**DOI:** 10.1128/genomeA.00064-14

**Published:** 2014-03-06

**Authors:** Y. M. Wong, J. C. Juan, H. M. Gan, C. M. Austin

**Affiliations:** a School of Science, Monash University Malaysia, Jalan Lagoon Selatan, Bandar Sunway, Malaysia; b Nanotechnology & Catalysis Research Centre (NANOCAT), University of Malaya, Kuala Lumpur, Malaysia

## Abstract

*Clostridium perfringens* strain JJC is an effective biohydrogen and biochemical producer that was isolated from landfill leachate sludge. Here, we present the assembly and annotation of its genome, which may provide further insights into the gene interactions involved in efficient biohydrogen production.

## GENOME ANNOUNCEMENT

Hydrogen is a promising clean alternative to fossil fuel (1, 2). It is an environmentally safe energy source that produces water as the only end product and hence will not induce air pollution and climate change. Hydrogen can be biologically produced from organic rich waste via fermentation (3–5). Therefore, biohydrogen production is an environmentally friendly approach that converts waste into energy.

Hydrogen-producing bacteria are naturally found in the environment (6–9). The isolation of hydrogen-producing bacteria from waste is of particular interest because they can survive and tolerate external stress. In our work, we attempted to isolate efficient hydrogen-producing bacteria from landfill leachate sludge. This sludge originated from a sanitary landfill, and hence, it carries a microbial community similar to that of the landfill. Hydrogen-producing bacteria that live in these environments can adapt to harsh living conditions and have unique properties in the degradation of organic waste and the production of biohydrogen and biochemicals.

*Clostridium perfringens* is a Gram-positive and spore-forming strict anaerobe. It can ferment a vast range of carbohydrates and produce by-products, including acetate, butyrate, lactate, ethanol, hydrogen, and carbon dioxide, which have industrial applications (10). The ability of *C. perfringens* strain JJC to produce biohydrogen is of great value to the field of renewable energy production, and hence we sequenced its genome to (i) identify genes that inhibit and promote hydrogen production and (ii) aid in the future of cloning and metabolic engineering of this strain.

The genome sequencing of strain JJC was performed using the Illumina MiSeq benchtop sequencer (2 × 150-bp paired-end sequencing). The reads were trimmed and assembled *de novo* using the CLC Genomics Workbench 6.0 (CLC bio, Denmark). Multiple-genome alignment was conducted using Gegenees 2.0.3. The average similarities of the conserved core and the size of core were set at 20% (11). The genome sequence was annotated with the Rapid Annotations using Subsystems Technology (RAST) server (12). RNAmmer 1.2 and tRNAscan-SE 1.21 were used to predict rRNAs and tRNAs, respectively (13, 14). Based on 16S rRNA analysis, strain JJC has a 100% identity score to *C. perfringens* ATCC 13124 and 99% to *C. perfringens* strains 13 and SM101. In addition, the heat plot from multiple-genome alignment revealed that strain JJC shares 95% similarities to strains 13 and ATCC 13124 and 88% to strain SM101. The results proved that strain JJC is a new strain of *C. perfringens*. The draft genome sequence of strain JJC comprises 3,259,329 bases in 69 contigs. It has a G+C content of 28.12% and contains 2,986 genes, 5 rRNAs, and 67 tRNAs.

*C. perfringens* JJC contains two hydrogenases: [Fe] hydrogenase HydA and a dimeric cytoplasmic [Fe] hydrogenase. These proteins are activated and modified by three [FeFe]-hydrogenase maturation proteins, namely, HydE, HydF, and HydG (15, 16). In addition, it contains genes encoding products such as butyrate kinase (17) and acetate kinase (18) that are involved in the production of organic acids and solvents, including butyrate and acetate.

### Nucleotide sequence accession numbers.

This whole-genome shotgun project has been deposited at DDBJ/EMBL/GenBank under the accession no. AWRZ00000000. The version described in this paper is version AWRZ01000000.
